# Effect of Pharmacogenetics on Renal Outcomes of Heart Failure Patients with Reduced Ejection Fraction (HFrEF) in Response to Dapagliflozin

**DOI:** 10.3390/pharmaceutics17080959

**Published:** 2025-07-24

**Authors:** Neven Sarhan, Mona F. Schaalan, Azza A. K. El-Sheikh, Bassem Zarif

**Affiliations:** 1 Clinical Pharmacy Department, Faculty of Pharmacy, Misr International University, Cairo 11314, Egypt; mona.schaalan@miuegypt.edu.eg; 2 Basic Health Sciences Department, College of Medicine, Princess Nourah bint Abdulrahman University, P.O. Box 84428, Riyadh 11671, Saudi Arabia; aaelsheikh@pnu.edu.sa; 3 Cardiology Department, National Heart Institute, Ministry of Health and Population, Cairo 11796, Egypt; bassemzarif@gmail.com

**Keywords:** heart failure, dapagliflozin, polymorphism, cardiac fibrosis, renal outcome, precision medicine

## Abstract

**Background/Objectives**: Heart failure with reduced ejection fraction (HFrEF) is associated with significant renal complications, affecting disease progression and patient outcomes. Sodium-glucose co-transporter-2 (SGLT2) inhibitors have emerged as a key therapeutic strategy, offering cardiovascular and renal benefits in these patients. However, interindividual variability in response to dapagliflozin underscores the role of pharmacogenetics in optimizing treatment efficacy. This study investigates the influence of genetic polymorphisms on renal outcomes in HFrEF patients treated with dapagliflozin, focusing on variations in genes such as *SLC5A2*, *UMOD*, *KCNJ11*, and *ACE*. **Methods**: This prospective, observational cohort study was conducted at the National Heart Institute, Cairo, Egypt, enrolling 200 patients with HFrEF. Genotyping of selected single nucleotide polymorphisms (SNPs) was performed using TaqMan™ assays. Renal function, including estimated glomerular filtration rate (eGFR), Kidney Injury Molecule-1 (KIM-1), and Neutrophil Gelatinase-Associated Lipocalin (NGAL) levels, was assessed at baseline and after six months of dapagliflozin therapy. **Results**: Significant associations were found between genetic variants and renal outcomes. Patients with AA genotype of rs3813008 (*SLC5A2*) exhibited the greatest improvement in eGFR (+7.2 mL ± 6.5, *p* = 0.004) and reductions in KIM-1 (−0.13 pg/mL ± 0.49, *p* < 0.0001) and NGAL (−6.1 pg/mL ± 15.4, *p* < 0.0001). Similarly, rs12917707 (*UMOD*) TT genotypes showed improved renal function. However, rs5219 (*KCNJ11*) showed no significant impact on renal outcomes. **Conclusions**: Pharmacogenetic variations influenced renal response to dapagliflozin in HFrEF patients, particularly in *SLC5A2* and *UMOD* genes. These findings highlighted the potential of personalized medicine in optimizing therapy for HFrEF patients with renal complications.

## 1. Introduction

Heart failure (HF) is a major public health concern worldwide, with an estimated 64.3 million individuals affected [1]. According to the Heart Failure Society of America (HFSA), over 6.7 million American adults are currently suffering from HF. This figure is anticipated to rise to 8.7 million by 2030, 10.3 million by 2040, and a whopping 11.4 million by 2050. HF is also regarded as one of the most common hospitalization reasons and is associated with high incidence of morbidity and mortality [2]. The HFSA also states that HF was a contributory factor in more than 425 thousand fatalities, accounting for 45% of cardiovascular deaths in the United States in 2021 [3].

Sodium-glucose co-transporter-2 inhibitors (SGLT2is), initially developed to manage blood glucose levels in patients with type 2 diabetes, are now considered one of the latest therapeutic strategies for HF as outlined in the 2022 American Heart Association (AHA) guidelines [4]. This class, which includes empagliflozin, canagliflozin, and dapagliflozin, has been shown to significantly reduce hospitalizations for heart failure [4].

Dapagliflozin inhibits SGLT2, a transporter protein encoded by the SLC5A2 gene. Mutations in SLC5A2 can alter the expression, membrane localization, or function of SGLT2, potentially altering drug efficacy [5]. During metabolism, dapagliflozin primarily undergoes glucuronidation by the uridine 5′-diphosphate glucuronosyltransferase (UGT) isomer UGT1A9, with additional metabolism by UGT2B4 and cytochrome P450 3A4 [6,7].

Genetic polymorphisms in glucose transport, renal, and metabolic control genes may form part of the explanation for between-subject variation in response to SGLT2 inhibitors. The SLC5A2 gene product is sodium-glucose co-transporter 2 (SGLT2), the predominant pharmacological target of dapagliflozin. These alleles such as rs3813008 have been associated with an alteration of transporter function, which may have implications on glucose reabsorption and drug action [8,9]. The UMOD gene bearing the typical rs12917707 encodes for uromodulin—a protein related to tubular sodium handling and kidney protection. The variant was linked with variation in uromodulin expression and chronic kidney disease risk [10,11] and thus indirectly influenced therapeutic outcomes following SGLT2 inhibitor treatment.

KCNJ11, linked with the rs5219 variant (E23K), codes a subunit of the pancreatic β-cell ATP-sensitive potassium channel. This polymorphism has been indicated to affect insulin secretion and, in addition, has become pharmacokinetically relevant in type 2 diabetes [12,13]. Finally, the ACE gene, as tagged by rs4343, is also heavily involved in the renin–angiotensin–aldosterone system (RAAS), a regulator of renal hemodynamics and blood pressure. This polymorphism has been linked to variation in circulating levels of ACE and may influence hemodynamic responses to SGLT2 inhibitors [14,15]. Variations in the genes encoding these enzymes may alter dapagliflozin pharmacokinetics, leading to differences in plasma exposure and therapeutic response [16].

The development of SGLT2i represents a significant advancement in managing HFrEF. These drugs not only improve cardiovascular outcomes but also provide substantial renal protection, as demonstrated in several landmark trials such as DAPA-CKD and CREDENCE [17,18].

Despite these benefits, individual responses to SGLT2i vary, highlighting the importance of pharmacogenetic studies to demonstrate genetic variations influencing drug response. Genetic polymorphisms in key genes, as SLC5A2 and APOL1, may account for this variability by modulating the drugs’ mechanisms of action, including effects on tubule–glomerular feedback, renal hemodynamics, and metabolic pathways [7,16,19]. Identifying these pharmacogenetic markers could enhance personalized medicine approaches, optimizing therapy for HFrEF patients and minimizing dapagliflozin adverse effects.

Apart from its potential clinical significance, exploring the pharmacogenetics of SGLT2i can provide insights into the molecular mechanisms underlying HFrEF and related renal symptoms. Exploring gene–drug interactions may facilitate the identification of novel therapeutic targets and improve the prediction of treatment outcomes. This is particularly crucial for populations with diverse genetic backgrounds, as variations in allele frequencies and genetic risk factors can influence treatment efficacy and safety [7,20]. Addressing these gaps, our study seeks to advance precision medicine and promote equitable healthcare.

The goal of this study was to investigate the role of genetic variants in predicting renal outcomes in HFrEF patients receiving dapagliflozin, focusing on interindividual variation in response to medication by investigating the relationships between certain single nucleotide polymorphisms (SNPs) and renal outcomes. Furthermore, the study investigated the effect of these genetic markers on cardiovascular outcomes and adverse events, contributing to a deeper understanding of pharmacogenetics in this context.

## 2. Materials and Methods

### 2.1. Study Design and Patients’ Population

**Figure 1:**
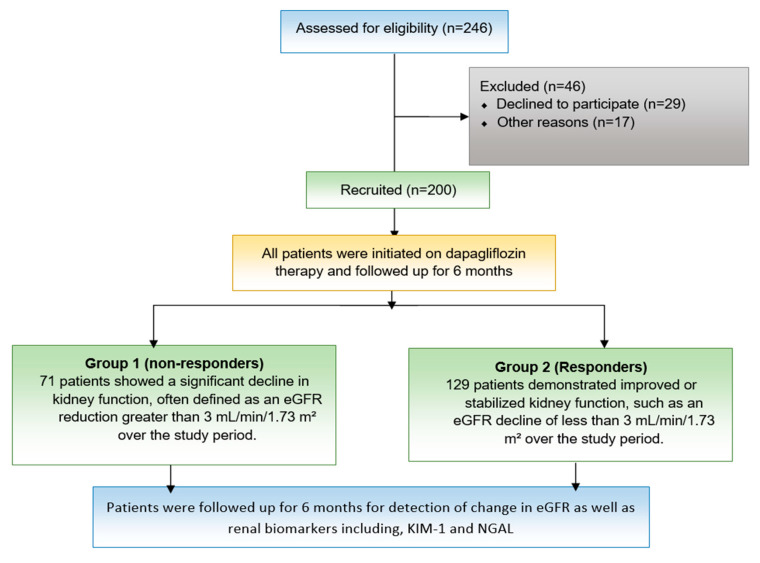
Study flow chart. A schematic representation of the study design, including patient eligibility assessment, recruitment, exclusion criteria, and follow-up. A total of 246 patients were assessed, with 200 recruited and 46 excluded for various reasons.

This study was a prospective, observational cohort design that was conducted in cardiology clinics at the National Heart Institute, Cairo, Egypt. The study spanned 1.5 years, including a 12-month recruitment and data analysis phase and a 6-month follow-up for each participant, as shown in Figure 1. The study was registered on ClinicalTrial.gov registry (Trial identifier NCT06901609) and was approved by the Organization of Teaching Hospitals and Institutes (GOTHI) Research Ethics Committee, Cairo, Egypt (Institutional Review Board (IRB) no. IHC00068). All patients provided written informed consent prior to participation.

The inclusion criteria were adults (≥18 years) with HFrEF (left ventricular ejection fraction (LVEF) ≤40%) initiating dapagliflozin while on stable background HF therapy for at least four weeks. Exclusion criteria included hypersensitivity to dapagliflozin, a history of ketoacidosis or recurrent urinary tract infections, end-stage renal disease (estimated glomerular filtration rate; eGFR of <30 mL/min/1.73 m^2^), life expectancy under six months due to non-cardiovascular conditions, pregnancy, and lactation. A total of 200 participants were recruited, based on sample size calculations using G power software (version 3.1) that provided 80% power to detect significant genetic associations.

### 2.2. Study Protocol

Clinical and medical data were extracted from the electronic medical record, encompassing baseline demographics, NYHA classification, smoking status, medical history, and renal function metrics such as eGFR and albuminuria, in addition to information on concomitant treatments. During follow-up, renal outcomes (e.g., changes in eGFR, incidence of renal replacement therapy, and albuminuria levels), cardiovascular outcomes (e.g., heart failure hospitalizations and major adverse cardiac events), and adverse effects of dapagliflozin were documented. Whole blood samples (7 mL) were obtained for DNA extraction and genotyping. Evaluation of echocardiographic findings and assessment of kidney function tests were performed and repeated after 6 months.

The assessment of Standard Transthoracic 2D and Doppler echocardiography were performed using a Siemens ACUSON SC2000 system (Munich, Germany). Left ventricular volumes and LVEF were measured using the Simpson biplane method from apical four-chamber and apical two-chamber images, with LVEF indexed to body surface area [21]. To minimize variability in equipment and interpretation, the same cardiologist analyzed all images at both the beginning and end of the study using the same equipment.

### 2.3. Genotyping

Genomic DNA was extracted from blood samples (500 µL) using the standard phenol chloroform procedure. NanoDrop 2000/2000c spectrophotometer was used to measure DNA quality and concentration (Thermo Fisher Scientific, Waltham, MA, USA). For genotyping the specified SNPs (SLC5A2 rs3813008, KCNJ11 rs5219, UMOD rs12917707, and ACE rs4343), TaqMan™ SNP Genotyping Assays from Thermo Fisher were utilized (ID assay: C__27487769_10, C__11654065_10, C__31122302_20 and C_11942562_20 (Thermo Fisher Scientific, Waltham, MA, USA). All procedures used 10 μL of final volume and 10 ng genomic DNA. Allelic discrimination was performed using the StepOne RT-PCR apparatus and StepOne v2.0 software (Applied Biosystems, Waltham, MA, USA), as well as conventional qRT-PCR cycle settings.

### 2.4. Biochemical Assay

For measuring Kidney Injury Molecule-1 (KIM-1) and Neutrophil Gelatinase-Associated Lipocalin (NGAL), commercially available enzyme-linked immunosorbent assay (ELISA) kits were utilized. KIM-1 levels were assessed using the SMC™ KIM-1 Immunoassay Kit (Assay ID: C_03-0118-00) (EMD Millipore, Billerica, MA, USA). NGAL was measured using the Human NGAL ELISA Kit (Catalog Number: KIT 036CE, BioPorto Diagnostics, Hellerup, Denmark).

### 2.5. Renal Response to Dapagliflozin

Renal responses to SGLT2i refer to the effect these medications have on kidney function, particularly in patients with chronic kidney disease (CKD) or diabetes. A positive renal response is typically characterized by an initial decline in estimated eGFR, followed by stabilization or slowed progression of kidney function decline over time. This response is associated with reduced albuminuria, lower risk of acute kidney injury, and long-term renal protection [22,23].

Criteria for renal response were as follows: Responders demonstrated improved or stabilized kidney function, such as an eGFR decline of less than 3 mL/min/1.73 m^2^ per year, along with reduced albuminuria and decreased progression of CKD, while non-responders showed a significant decline in kidney function, defined as an eGFR reduction greater than 5 mL/min/1.73 m^2^ over the study period or worsening albuminuria despite treatment [23,24], as shown in Figure 1.

### 2.6. Sample Size Calculation

Sample size estimation was conducted to ensure adequate statistical power for detecting significant associations between genetic polymorphisms and renal outcomes in HFrEF patients treated with dapagliflozin. Power analysis was performed using G*Power software version 3.1, targeting a statistical power of 80% (β = 0.20) and a significance level of 0.05, adjusted using Bonferroni correction for multiple comparisons. Based on prior studies, such as [10,14,15], which investigated the effects of SGLT2i on renal function, an effect size (Cohen’s f) of 0.25 was assumed for one-way ANOVA, requiring a minimum of 159 participants to detect meaningful differences among genotype groups. To account for potential dropouts and missing data, an additional 20% of participants were included, bringing the total recruitment target to 200 patients. This sample size was deemed sufficient to detect differences in eGFR changes, KIM-1, and NGAL levels across genetic groups while maintaining statistical robustness and minimizing the risk of Type I and Type II errors.

### 2.7. Data Analysis

The statistical analysis in this study employed various tests to evaluate the impact of genetic polymorphisms on renal outcomes in HFrEF patients treated with dapagliflozin. Descriptive statistics, including means and standard deviations, were used to summarize baseline demographic and clinical characteristics. Normality testing was performed using the Shapiro–Wilk test to determine whether continuous variables followed a normal distribution. Group comparisons between responders and non-responders were conducted using independent *t*-tests for normally distributed continuous variables, Mann–Whitney U tests for non-normally distributed data, and chi-square tests for categorical variables.

Hardy–Weinberg equilibrium (HWE) was assessed using chi-square tests to evaluate genotype distributions. The association between genetic variants and renal function changes (eGFR, KIM-1, and NGAL levels) was analyzed using one-way ANOVA, followed by post hoc tests to identify significant differences across genotypes. To correct multiple comparisons and reduce the risk of Type I errors, Bonferroni correction was applied where appropriate.

Multivariable regression models were utilized to determine the independent effect of SNPs on renal outcomes, adjusting for potential confounders such as age, sex, and baseline eGFR. All statistical analyses were conducted using SPSS software version 20, with significance set at *p* < 0.05.

## 3. Results

### 3.1. Baseline Characteristics of the Study Cohort

From November 2023 to December 2024, a total of 246 patients were evaluated for eligibility, with 200 meeting the inclusion criteria and later enrolling at the National Heart Institute in Cairo, Egypt. The baseline demographic, clinical, and laboratory characteristics of the study participants are presented in Table 1. There were no significant differences between responders and non-responders regarding age, sex, prevalence of diabetes mellitus, hypertension, or complete blood count parameters. Similarly, liver and kidney function tests, along with echocardiographic parameters (ejection fraction, end systolic volume, and end diastolic volume), showed no statistically significant differences between the groups. Baseline levels of biochemical markers, including KIM-1 and NGAL, were also comparable between groups (*p* > 0.05).

The genotype frequencies of all tested SNPs were consistent with Hardy–Weinberg equilibrium, indicating a representative sample (Table S1). The minor allele frequencies (MAFs) observed were 0.41 for rs3813008 (SLC5A2), 0.24 for rs5219 (KCNJ11), 0.33 for rs12917707 (UMOD), and 0.46 for rs4343 (ACE).

### 3.2. Comparison Between Responders and Non-Responders Regarding Post Treatment Assessed Outcome and Their Respective Genotypes

Responders showed a significant improvement in eGFR compared to non-responders (82.9 ± 18.6 vs. 73.6 ± 20.3, respectively, *p* = 0.014), with a mean increase of 9.9 ± 2.9 in responders and a decline of −2.9 ± 4.2 in non-responders (*p* < 0.0001). Notably, reductions in KIM-1 and NGAL levels were observed among responders compared to non-responders (*p* < 0.001), suggesting improved renal function and decreased renal injury in the responder group as presented in Table 2.

### 3.3. Association Between SNPs and Renal Response

Regarding SNP rs3813008 (SLC5A2), individuals with the AA genotype exhibited the greatest improvement in eGFR (7.2 ± 6.5, *p* = 0.004) and the most pronounced reduction in KIM-1 levels (−0.13 ± 0.49, *p* < 0.0001). Additionally, the AA genotype was associated with a significant decrease in NGAL levels (−6.1 ± 15.4, *p* < 0.0001). In contrast, those with the GG genotype showed a decline in eGFR (−2.6 ± 3.6) and an increase in NGAL (73.3 ± 18.5). This suggests a protective effect of the A allele in renal function preservation, as shown in Table 2 and Figure S1.

Similarly, for SNP rs12917707 (UMOD), participants with the TT genotype demonstrated a significant increase in eGFR (6.3 ± 3.7, *p* = 0.017) and a notable reduction in KIM-1 (−0.11 ± 0.19, *p* = 0.01). Conversely, the CC genotype was linked to negligible changes in renal markers, underscoring the potential influence of this SNP on renal response to treatment, as shown in Table 2 and Figure S2. In addition, for SNP rs4343 (ACE), the GG genotype was associated with the most significant improvement in renal function, evidenced by an increase in eGFR (−0.71 ± 1.2, *p* = 0.004) and substantial decreases in both KIM-1 (−0.37 ± 0.18, *p* < 0.0001) and NGAL levels (−46.9 ± 6.3, *p* = 0.002) (Table 2 and Figure S3). However, for SNP rs5219 (KCNJ11), this SNP did not significantly affect renal outcomes, as indicated by non-significant changes in eGFR (*p* = 0.74) and biochemical markers (*p* > 0.05), as shown in Table 3 and Figure S4.

### 3.4. Predictors of Renal Response

**Figure 2:**
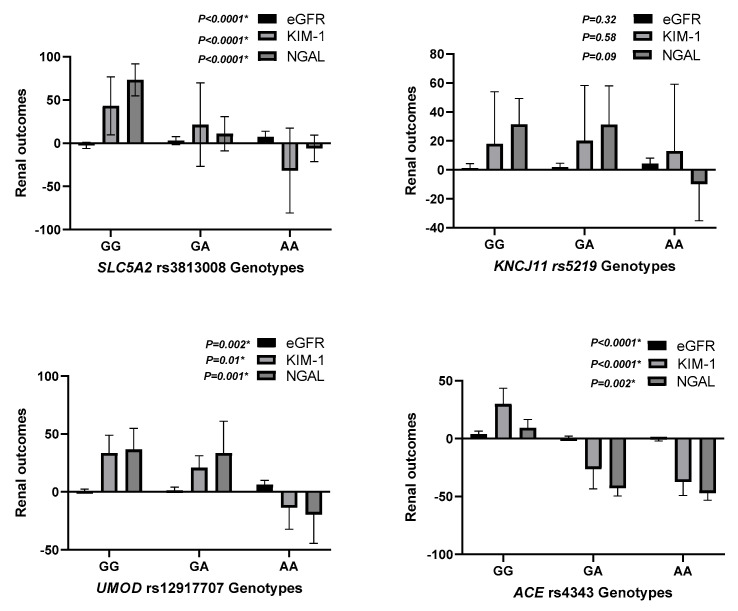
Predictors of Renal Response to Dapagliflozin.

Multiple regression analysis (Table 4 and Figure 2) identified rs3813008 and rs12917707 as independent predictors of variability in renal response to dapagliflozin. The rs3813008 (SLC5A2) showed a strong positive association with changes in eGFR (β = 4.7 ± 0.78, *p* = 0.001) and a negative association with changes in KIM-1 (β = −0.26 ± 0.1, *p* < 0.0001) and NGAL (β = −0.42 ± 1.76, *p* = 0.0001), indicating improved renal function and reduced kidney injury among carriers of the A allele. Moreover, rs12917707 (UMOD) is associated with moderate improvements in renal function, particularly reflected in changes in eGFR (β = 1.6 ± 0.78, *p* = 0.043) and reductions in KIM-1 (β = −0.13 ± 0.1, *p* = 0.017), suggesting a potentially beneficial role of this polymorphism in enhancing renal outcomes.

This figure summarizes the key predictors of renal response to dapagliflozin therapy based on multivariable regression analysis. Genetic polymorphisms in *SLC5A2* (rs3813008) and *UMOD* (rs12917707) were identified as significant determinants of changes in eGFR, KIM-1, and NGAL levels. These findings emphasize the role of pharmacogenetics in optimizing renal outcomes in heart failure patients receiving Dapagliflozin. eGFR, estimated glomerular filtration rate; KIM-1, Kidney Injury Molecule-1; NGAL, Neutrophil Gelatinase-Associated Lipocalin.

Moreover, in multivariate logistic regression, we evaluated the correlation of selected SNPs and clinical variables with response. Among the four SNPs under study, rs3813008 (SLC5A2) and rs1291770 (UMOD) were found to have significant correlations with response status. Specifically, rs3813008 showed a high association (β = 1.922, *p* = 3 × 10^−6^), with an odds ratio (OR) of 6.8 (95% CI: 2.9–15.7), reflecting a strongly increased risk of response in carriers of the variant allele. Similarly, rs1291770 was also significantly associated with therapeutic response (β = 0.779, *p* = 0.011), with an OR of 2.2 (95% CI: 1.2–4.1). On the other hand, rs5219 (KCNJ11) and rs4343 (ACE) did not correlate significantly with response. No clinical covariates achieved statistical significance, but non-significant trends appeared in variables such as diabetes and sex, as shown in Table S3.

The impact of genetic variation on kidney function and biomarker response to dapagliflozin were assessed in Table S4. We examined associations of individual SNPs with baseline kidney measurements and changes in them over time. Rs3813008 (SLC5A2) showed a significant association with higher baseline eGFR (β = 9.1, *p* = 0.003), treatment improvement in eGFR (β = 5.1, *p* < 0.00001), and reductions in both KIM-1 (β = −0.276, *p* = 0.00003) and NGAL (β = −23.7, *p* = 0.00007) levels, indicating improved tubular as well as glomerular function. Similarly, rs1291770 (UMOD) was associated with higher baseline eGFR (β = 7.6, *p* = 0.001), larger eGFR response (β = 1.98, *p* = 0.006), and notable declines in KIM-1 (β = −0.12, *p* = 0.021) and NGAL (β = −19.7, *p* = 0.005). In addition, rs1291770 was related to lowered baseline concentrations of KIM-1 (β = 0.57, *p* = 0.009). In contrast, no significant associations were found in models with rs5219.

In additional exploratory analysis (Table S5), we explored possible modifying effects of genetic variants on other treatment outcomes. As noted before, rs3813008 (SLC5A2) remained a strong predictor of increased response, with significant effects on multiple parameters. Likewise, rs1291770 (UMOD) had repeatable associations with positive kidney-related outcomes. None of rs5219 (KCNJ11) or rs4343 (ACE) had an effect within this expanded model. These results validate the functional importance of SLC5A2 and UMOD gene variants in modulating patient response to SGLT2i therapy and suggest their potential as predictive biomarkers in the clinic.

### 3.5. Correlation Between Renal Biomarkers and eGFR

Results showed a strong negative correlation between eGFR and both KIM-1 (r = −0.764, *p* < 0.0001) and NGAL (r = −0.722, *p* < 0.0001), indicating that worsening renal function is associated with increased levels of biomarkers, as shown in Table 4. Additionally, KIM-1 and NGAL exhibited a strong positive correlation (r = 0.714, *p* < 0.0001), suggesting that they might tend to concomitantly increase. In contrast, the correlation between eGFR and LVEF was weak and not statistically significant (r = −0.140, *p* = 0.09), as was the relationship between KIM-1 and LVEF (r = −0.011, *p* = 0.884) and NGAL and LVEF (r = 0.061, *p* = 0.416). These findings suggested that renal dysfunction, as indicated by declining eGFR, was strongly linked to increases in KIM-1 and NGAL levels, while LVEF did not show a significant association with renal biomarkers in HF patients, as presented in Table 5.

## 4. Discussion

The aim of the current study was to investigate the impact of genetic polymorphisms on renal responses to dapagliflozin in a cohort of Egyptian patients. To the best of the authors’ knowledge, this study is the first attempt to examine the interindividual genetic impact on kidney function outcomes of using dapagliflozin in HFrEF patients. The results demonstrated significant associations between specific SNPs and improvements in renal functions, as indicated by changes in eGFR and reductions in renal injury biomarkers, including KIM-1 and NGAL. These findings highlighted valuable insights into the pharmacogenomics of dapagliflozin and its potential role in preserving kidney functions among HFrEF patients.

One of the most notable findings was the strong association between the rs3813008 SNP in the SLC5A2 gene and renal outcomes following dapagliflozin therapy. Individuals carrying the AA genotype demonstrated the most significant improvements in eGFR (+7.2 ± 6.5, *p* = 0.004) and marked reductions in KIM-1 (−0.13 ± 0.49, *p* < 0.0001) and NGAL (−6.1 ± 15.4, *p* < 0.0001). In contrast, those with the GG genotype exhibited a decline in eGFR and an increase in NGAL levels. These findings suggest that the A allele might confer a protective effect against renal dysfunction in response to dapagliflozin.

The SLC5A2 gene encodes the SGLT2 transporter, which plays a crucial role in glucose reabsorption in the renal proximal tubules. Variants in this gene can alter transporter activity and influence the efficacy of dapagliflozin. Genetic variations have previously been reported to modify protein and transporter function in dapagliflozin’s mechanism of action [8,9,19]. However, as rs3813008 is an intronic SNP, the mechanism by which it may influence SGLT2 function is unknown. One possible reason is that the SNP reduces SGLT2 function, acting similar to a natural SGLT2i. As a result, when these patients are treated with a transporter-blocking medication, they profit slightly (if at all) more than other patients. Additional functional studies of this variant might reveal the potential mechanism by which this SNP affects outcomes in HF [9,19].

Previous studies have reported similar findings, linking genetic variations in SLC5A2 to differential responses to dapagliflozin. These results highlighted the importance of SLC5A2 polymorphisms in modulating renal function and suggested that genetic screening could help identify patients who were more likely to benefit from SGLT2i therapy [7]. Moreover, previous research indicated a possible link between rs3813008 and insulin levels during an oral glucose tolerance test (OGTT), specifically at the 30 min mark. Additionally, this SNP was associated with changes in systolic blood pressure over time [7], although the findings were not consistent across different studies [5,16]. Despite these observations, the overall impact on glycemic control and disease risk remains inconclusive, highlighting the need for further research to determine its clinical significance [7].

The current findings revealed the positive association of the rs12917707 SNP in the UMOD gene with promising improvements in kidney function outcomes with dapagliflozin. Patients carrying the TT genotype exhibited a significant increase in eGFR (+6.3 ± 3.7, *p* = 0.017) and decline in KIM-1 levels (−0.11 ± 0.19, *p* = 0.01), suggesting a protective effect against kidney injury. The gene encodes uromodulin, a protein essential for kidney function and ion transport regulation. Genetic variations in UMOD have been associated with susceptibility and progression of CKD [5]. A previous study has shown that UMOD polymorphisms impact tubular function and sodium handling, potentially influencing the therapeutic response to SGLT2i [5]. The present study is in alignment with the latter findings, indicating that UMOD variants may serve as predictive markers for renal outcomes in patients treated with SGLT2i. Additional research is warranted to better unveil the underlying mechanisms of these associations and their clinical significance.

In contrast to rs3813008 and rs12917707, the rs5219 SNP in the KCNJ11 gene was not significantly associated with improved renal outcomes. The distribution of genotypes (CC, CT, and TT) revealed no significant differences in eGFR changes (*p* = 0.74) or biochemical markers (*p* > 0.05) assessed in the current study. KCNJ11 encodes the ATP-sensitive potassium (K-ATP) channel subunit Kir6.2, which regulates insulin secretion and glucose metabolism. Despite the reported association of KCNJ11 polymorphisms with increased risk of type 2 diabetes and pharmacogenetic responses to antidiabetic medications, their role in the regulation of kidney function appears to be minor [25]. The latter insignificant associations, revealed in our study findings, suggested that KCNJ11 variants did not directly affect the renal protective effects of SGLT2i, despite their reported impact on metabolic outcomes. This finding highlighted the specificity of certain genetic determinants in regulating drug response and emphasized the importance of conducting targeted pharmacogenomic studies across diverse patient populations.

The rs4343 SNP in the ACE gene was significantly associated with improved renal function among individuals with the GG genotype. This group exhibited the greatest increase in eGFR (−0.71 ± 1.2, *p* = 0.004) and substantial reductions in KIM-1 (−0.37 ± 0.18, *p* < 0.0001) and NGAL (−46.9 ± 6.3, *p* = 0.002). The ACE gene encodes angiotensin-converting enzyme, a key regulator of the renin–angiotensin–aldosterone system (RAAS), which plays a critical role in blood pressure control and kidney function. Previous research demonstrated that ACE polymorphisms influenced susceptibility to CKD and response to RAAS inhibitors [26]. The current findings suggested that ACE variants might also impact the renal effects of dapagliflozin, potentially through interactions with the RAAS pathway. The precise mechanisms by which ACE polymorphisms modulated dapagliflozin renal response warrant further investigation.

The current study showed a significant negative correlation between eGFR and both KIM-1 and NGAL irrespective of changes in LVEF. These results were in concordance with the results of the Peralta et al., 2012 study, which examined the association between urinary KIM-1 and NGAL levels and the development of CKD among atherosclerotic patients, concluding that elevated levels of these biomarkers were linked to an increased risk of CKD progression [27]. Similarly, research by Latoch et al. (2020) evaluated KIM-1 and NGAL as markers of tubular injury in acute lymphoblastic leukemia survivors, demonstrating their effectiveness in detecting acute kidney injury and highlighting their potential utility in monitoring nephrotoxicity during chemotherapy [28]. Both studies in addition to several other studies underscored the role of KIM-1 and NGAL as early indicators of kidney dysfunction across different patient populations [27,28,29].

### Clinical Implications and Future Directions

The findings, presented in the current study, emphasized the potential of implementing pharmacogenomics to advance personalized medicine in fields of nephrology and cardiology. The identification of genetic markers regulating better renal responses to dapagliflozin could facilitate more individualized treatment strategies, enhancing efficacy and minimizing the risk of adverse effects. Nevertheless, several limitations should be noted. First, the limited sample size and population specificity were due to the single-center study setting on Egyptian patients’ cohort. Therefore, larger, multi-ethnic studies are warranted to confirm these results and to investigate population-specific genetic influences. The second limitation is the lack of functional analysis, while significant genetic associations were identified. Thus, further functional studies are needed to clarify the biological mechanisms behind these effects. The lack of a comparator group in this prospective observational study limits inferring causality since renal improvement observed may be due to natural course of disease or the action of concomitant treatments of heart failure. Follow-up randomized pharmacogenetic studies with adequate control arms are warranted to further separate and establish the direct effects of SGLT2 inhibitor treatment. Moreover, there is a lack of assessment of the polymorphic expression of UGT1A9, which may significantly influence the pharmacokinetic variability in the elimination of dapagliflozin. The presence of potential confounding factors is the latter limitation; although baseline characteristics were accounted for, factors such as medication adherence, lifestyle differences, and comorbidities might still impact renal outcomes. Future research should consider integrating pharmacogenomic screening into clinical practice and explore additional genetic variants that may impact SGLT2i response. By combining genetic data with clinical and biochemical parameters, a more comprehensive understanding of interindividual variability in drug response could be achieved, ultimately enhancing patient care and outcomes.

## 5. Conclusions

Specific genetic polymorphisms, especially in the SLC5A2 and UMOD genes, might be linked to varying renal responses to dapagliflozin. While these preliminary findings suggest a potential role of genetics in influencing drug efficacy, they should be interpreted with caution. Further large-scale and mechanistic studies are needed to validate these associations and to better understand their possible clinical implications for personalized treatment strategies in patients with HFrEF and CKD.

## Figures and Tables

**Table 1:** Baseline patient demographics and laboratory values in both groups 1 and 2.

Characteristics	Non-Responder Group (n = 71)	Responder Group (n = 129)	Significance (*p*-Value)
Age (yrs); mean ± SD	55.6 ± 10.54	57.1 ± 11.1	0.48
Sex; n (%)			
Male	24 (33.8%)	43 (33.3%)	0.92
Female	47 (66.2%)	>86 (66.7%)	
Diabetes mellitus; n (%)	29 (40.8%)	49 (38%)	0.76
Hypertension; n (%)	30 (42.3%)	57 (44.2%)	0.67
Heart rate	82.6 ± 16.5	81.2 ± 16.7	0.58
CBC; mean ± SD			
RBCs (10^6^ cells/µL)	4.92 ± 0.81	4.85 ± 0.72	0.34
Hemoglobin (gm/dL)	12.9 ± 1.74	12.4 ± 1.66	0.96
WBCs (10^3^ cells/µL)	8.65 ± 2.6	8.49 ± 2.1	0.13
Platelets (10^3^ cells/µL)	259.2 ± 100.1	253.6 ± 77.9	0.29
Liver function Tests; mean ± SD			
AST (U/L)	23.4 ± 14.4	26.8 ± 18.2	0.18
ALT (U/L)	35.7 ± 11.6	30.1 ± 16.2	0.105
Kidney function Tests; mean ± SD			
BUN (mg/dL)	18.6 ± 8.9	21.3 ± 9.2	0.604
Serum creatinine (mg/dL)	1.08 ± 0.32	1.12 ± 0.41	0.19
Serum K (mEq/L); mean ± SD	4.1 ± 0.42	4.3 ± 0.58	0.09
eGFR	77.2 ± 22.9	73.1 ± 23.8	0.89
Echocardiographic parameters			
EF at baseline (%)	35.4 ± 11.2	35.3 ± 10.4	0.83
ESV at baseline (mL)	113.2 ± 42.3	116.2 ± 39.8	0.74
EDV at baseline (mL)	170.3 ± 72.1	164.6 ± 62.8	0.6
NYHA Class			
II	67 (51.9%)	38 (53.5%)	0.58
III	55 (42.6%)	27 (38%)	
IV	7 (5.4%)	6 (8.5%)	
Biochemical markers			
KIM-1 (pg/mL)	169.8 ± 71.4	174.2 ± 66.5	0.91
NGAL (pg/mL)	229.3 ± 99.6	248.7 ± 98.1	0.82

Statistical analysis: Values are means ± SD; significance is at *p* < 0.05. SD, standard deviation; CBC, complete blood count; RBCs, red blood cells; WBCs, white blood cells; AST, aspartate aminotransferase; ALT, alanine aminotransferase; BUN, blood urea nitrogen; K, potassium; EF, ejection fraction; ESV, end systolic volume; EDV, end diastolic volume; eGFR, estimated glomerular filtration rate; KIM-1, Kidney Injury Molecule-1; NGAL, Neutrophil Gelatinase-Associated Lipocalin.

**Table 2:** Patients’ laboratory values and biochemical markers 6-month post dapagliflozin therapy in both groups, 1 and 2.

Characteristics	Non-Responder Group	Responder Group	Significance (*p*-Value)
Kidney function Tests; mean ± SD			
- eGFR after 6 months	73.6 ± 20.3	82.9 ± 18.6	0.014 *
- eGFR difference	−2.9 ± 4.2	9.9 ± 2.9	0.0001 *
Echocardiographic parameters			
- EF after 6 months (%)	37.9 ± 12.6	38.9 ± 11.1	0.247
- ESV after 6 months (mL)	108.4 ± 27.1	101.3 ± 31.8	0.482
- EDV after 6 months (mL)	148.4 ± 51.8	140.8 ± 49.2	0.749
Biochemical markers			
- KIM-1 after 6 months (pg/mL)	224.4 ± 97.8	141.3 ± 58.3	<0.001 *
- Change in KIM-1 (pg/mL)	50.2 ± 31.1	−34.8 ±27.0	<0.0001 *
- NGAL after 6 months (pg/mL)	301.4 ± 132.9	196.4 ± 80.1	<0.001 *
- Change in NGAL (pg/mL)	72.1 ± 42.1	−52.4 ± 24.5	<0.001 *
*SLC5A2* SNP rs3813008			
- GG	0 (0%)	63 (48.8%)	0.0002 *
- GA	53 (73.2%)	57 (44.2%)	
- AA	18 (26.8%)	9 (7%)	
*KCNJ11* SNP rs5219			
- CC	40 (56.3%)	79 (61.2%)	0.119
- CT	22 (31%)	44 (34.1%)	
- TT	9 (12.7%)	6 (4.7%)	
*UMOD* SNP rs12917707			
- **GG**	29 (40.8%)	66 (51.2%)	0.001 *
- **GT**	24 (33.8%)	55 (42.6%)	
- **TT**	18 (25.4%)	8 (6.2%)	
*ACE* SNP rs4343			
- AA	16 (22.5%)	46 (35.7%)	0.0001 *
- AG	48 (67.6%)	45 (34.9%)	
- GG	7 (9.9%)	38 (29.5%)	

Statistical analysis: Values are means ± SD; * Significance is at *p* < 0.05. SD, standard deviation; EF, ejection fraction; ESV, end systolic volume; EDV, end diastolic volume; eGFR, estimated glomerular filtration rate; KIM-1, Kidney Injury Molecule-1; NGAL, Neutrophil Gelatinase-Associated Lipocalin.

**Table 3:** Renal parameters and biochemical markers comparison among different genotypes for rs3813008, rs5219, rs129177, and rs4343.

Treatment Group	eGFR After 6 Months (mL) Mean ± SD	Change in eGFR (mL) Mean ± SD	KIM-1 After 6 Months (pg/mL) Mean ± SD	Change in KIM-1 (pg/mL) Mean ± SD	NGAL After 6 Months (pg/mL) Mean ± SD	Change in NGAL (pg/mL) Mean ± SD
***SLC5A2* SNP rs3813008**						
**Non-Responders** **GG** **GA** **AA**	-------------	-------------	-------------	------------	-------------	-------------
	72.5 ± 24.5	−2.3 ± 4.4	210 ± 72	48 ± 33	280.8 ± 29.2	68.6 ± 31.9
	71.4 ± 25.2	−5.8 ± 2.9	205 ± 82	51 ± 32	339.9 ± 27.9	92.1 ± 33.7
**Responders** **GG** **GA** **AA**	71.1 ± 25.7	−3.1 ± 4.1	232 ± 87	52 ± 29	316.9 ± 36.5	72.4 ± 29.8
	79.9 ± 22.6	13.6 ± 0.65	143 ± 56	−38 ± 21	202.1 ± 14.8	−51.8 ± 24.6
	80.5 ± 21.9	9.9 ± 2.9	141 ± 58	−34 ± 17	196.3 ± 10.1	−52.4 ± 24.5
**Test Statistic**	F = 0.52	F = 161.4	F = 6.1	F = 144.5	F = 36.3	F = 345
***p*-Value**	0.133	<0.0001 *	<0.0001 *	<0.0001 *	0.001 *	<0.0001 *
***KCNJ11* SNP rs5219**						
**Non-Responders** **CC** **CT** ***TT***	75.6 ± 23.7	−3.1 ± 2.3	210 ± 80	49 ± 31	286.1 ± 41.3	74.2 ± 34.2
	68.9 ± 26.8	−1.8 ± 2.1	200 ± 92	45 ± 28	268.3 ± 26.3	71.7 ± 38.3
	65.3 ± 24.6	−3.9 ± 1.8	280 ± 42	66 ± 32	253.7 ± 61.4	50.5 ± 18.5
**Responders** **CC** **CT** ***TT***	71.6 ± 21.7	10.1 ± 2.9	151 ± 55	−32 ± 13	189.5 ± 16.8	−54.2 ± 26.8
	67.1 ± 22.1	9.7 ± 3.1	141 ± 60	−38 ± 26	206.7 ± 22.1	−49.9 ± 21.4
	74.5 ± 18.4	9.9 ± 2.7	144 ± 72	−34 ± 21	197.8 ± 17.9	−50.2 ± 22.6
**Test Statistic**	F = 0.71	F = 105.5	F = 5.2	F = 75.1	F = 3.7	F = 345
***p*-Value**	0.62	<0.0001 *	<0.0001 *	<0.0001 *	0.003 *	<0.0001 *
***UMOD* SNP rs12917707**						
**Non-Responders** **GG** **GT** **TT**	67.2 ± 20.5	−3.1 ± 4.2	220 ± 67	52 ± 37	286.8 ± 26.1	76.4 ± 19.3
	71.8 ± 30.1	−2.2 ± 3.9	190 ± 83	40 ± 21	274.9 ± 34.1	70.5 ± 40.5
	77.9 ± 25.1	−3.2 ± 4.8	240 ± 91	54 ± 34	232.8 ± 63.2	58.3 ± 36.1
**Responders** **GG** **GT** **TT**	67.5 ± 22.9	9.6 ± 2.9	150 ± 62	−35 ± 18	195.3 ± 37.8	−51.5 ± 23.4
	77.6 ± 20.9	10.1 ± 3.1	135 ± 53	−32 ± 17	196.2 ± 30.6	−52.3 ± 24
	80 ± 21.4	10.5 ± 2.8	138 ± 51	−36 ± 16	198.3 ± 33.5	−53.8 ± 28.2
**Test Statistic**	F = 0.45	F = 104	F = 5.3	F = 76.7	F = 3.8	F = 111.6
***p*-Value**	0.81	<0.0001 *	<0.0001 *	<0.0001 *	<0.003 *	<0.0001 *
***ACE* SNP rs4343**						
**Non-Responders** **AA** **AG** **GG**	68.6 ± 23.7	−2.8 ± 3.9	190 ± 72	43 ± 32	288.1 ± 38.9	75.1 ± 24.8
	68.8 ± 28.8	−2.9 ± 4.4	221 ± 41	50 ± 29	284.4 ± 25.4	72.5 ± 32.5
	72.6 ± 27.9	−2.5 ± 4.2	213 ± 78	46 ± 31	260.4 ± 32.9	69.5 ± 28.9
**Responders** **AA** **AG** **GG**	78.5 ± 23.2	9.9 ± 2.8	148 ± 56	−32 ± 16	186.6 ± 29.7	−44.6 ± 25
	70.3 ± 19.7	10.1 ± 3.3	140 ± 65	−35 ± 21	194.6 ± 15.8	−52.8 ± 23.5
	73.1 ± 22.7	10.3 ± 3.5	139 ± 60	−34 ± 17	232.9 ± 20.3	−69.8 ± 18.1
**Test Statistic**	F = 0.42	F = 102.4	F = 4.7	F = 73.8	F = 3.9	F = 112.3
***p*-Value**	0.83	<0.0001 *	<0.0001 *	<0.0001 *	0.002 *	<0.0001 *

Statistical analysis: Values are represented as means ± SD; (*) significantly different from baseline values is at *p* < 0.05; *p*-values are from ANOVA comparisons of means between genotypes for each renal outcome. eGFR, estimated glomerular filtration rate; KIM-1, Kidney Injury Molecule −1; NGAL, Neutrophil Gelatinase-Associated Lipocalin.

**Table 4:** Multiple regression analysis for predictors of renal outcomes among HFrEF patients in response to 6 months of dapagliflozin therapy.

Predictors of Renal Outcomes	Beta Coefficient ±SE	Adjusted R^2^	*p*-Value
**Change in eGFR**			
*SLC5A2* rs3813008 *SNP*	4.7 ± 0.78	0.49	0.001 *
*UMOD* rs12917707 *SNP*	1.6 ± 0.78		0.043 *
**Change in KIM-1**			
*SLC5A2* rs3813008 *SNP*	−26.2 ± 10.3	0.44	<0.0001 *
eGFR after 6 months	11.5 ± 4.2		0.002 *
*UMOD* rs1291770 *SNP*	−13.7 ± 11.6		0.017 *
**Change in NGAL**			
*SLC5A2* rs3813008 *SNP*	−42.5 ± 17.6	0.48	0.0001 *
*UMOD* rs12917707 *SNP*	−16.8 ± 12.3		0.032 *

* Significance is at *p* < 0.05 SNP, single nucleotide polymorphism; S.E, standard error; eGFR, estimated glomerular filtration rate; KIM-1, Kidney Injury Molecule-1; NGAL, Neutrophil Gelatinase-Associated Lipocalin.

**Table 5:** Correlation between renal biomarkers and eGFR among heart failure patients.

	Change in eGFR (mL/min)	Change in KIM-1 (ng/mL)	Change in NGAL (ng/mL)	Change in LVEF (%)
Change in eGFR (mL/min)		−0.764	−0.722	−0.140
		*p* < 0.0001 *	*p* < 0.0001 *	*p* = 0.09
Change in KIM-1 (ng/mL)	−0.764		0.714	−0.011
	*p* < 0.0001 *		*p* < 0.0001 *	*p* = 0.884
Change in NGAL (ng/mL)	−0.722	0.714		0.061
	*p* < 0.0001 *	*p* < 0.0001 *		*p* = 0.416
Change in LVEF (%)	−0.140	−0.011	0.061	
	*p* = 0.09	*p* = 0.884	*p* = 0.416	

* Data are given as r. Statistical analysis was carried out using Pearson’s correlation analysis. * Significant difference at *p* < 0.05; mL: milliliter; min: minute; ng: nanogram; eGFR: estimated glomerular filtration rate; KIM-1: Kidney Injury Molecule-1; NGAL: Neutrophil Gelatinase-Associated Lipocalin; r: Pearson’s correlation coefficient.

## Data Availability

The data presented in this study are available on request from the corresponding author.

## Supplementary Materials

The following supporting information can be downloaded at: https://www.mdpi.com/article/10.3390/pharmaceutics17080959/s1, Figure S1: Association Between rs3813008 (SLC5A2) Genotypes and Renal Outcomes; Figure S2: Association Between rs12917707 (UMOD) Genotypes and Renal Outcomes; Figure S3: Association Between rs4343 (ACE) Genotypes and Renal Outcomes; Figure S4: Lack of Association Between rs5219 (KCNJ11) Genotypes and Renal Outcomes; Table S1: Distribution of the studied genetic polymorphisms among study participants; Table S2: Patients’ laboratory values and biochemical markers 6-month post dapagliflozin therapy in both groups, 1 and 2; Table S3: Logistic Regression Analysis of SNPs and Clinical Predictors of Treatment Response; Table S4: Associations of SNPs with Baseline Kidney Parameters and Changes Over Time Post-SGLT2i; Table S5: Multivariate regression analysis for predictors of variability in clinical response to Dapagliflozin therapy among HFrEF patients.
