# Supplementary material for: Effect of Pharmacogenetics on Renal Outcomes of Heart Failure Patients with Reduced Ejection Fraction (HFrEF) in Response to Dapagliflozin
Source: Pharmaceutics. 2025 Jul 24;17(8):959. doi: 10.3390/pharmaceutics17080959 (PMC12389526; doi:10.3390/pharmaceutics17080959)
Supplement: Supplementary file 1 [file pharmaceutics-17-00959-s001.zip › pharmaceutics-3633641-supplementary.pdf]

Effect of Pharmacogenetics on Renal Outcomes of Heart Failure Patients with Reduced Ejection Fraction (HFrEF) in Response to dapagliflozin

Neven Sarhan [1,*], Mona F. Schaalan [1], Azza A.K. El-Sheikh [2] and Bassem Zarif [3]

1 Clinical Pharmacy Department, Faculty of Pharmacy, Misr International University, Cairo 11314, Egypt
2 Basic Health Sciences Department, College of Medicine, Princess Nourah bint Abdulrahman University, P.O. Box 84428, Riyadh 11671, Saudi Arabia
3 Cardiology Department, National Heart Institute, Ministry of Health and Population, Cairo, Egypt
* Corresponding author: Neven Sarhan. Adress: Villa 201 street 44, New Cairo, Cairo, Egypt, 11315. Email: nevine.mohamed@miuegypt.edu.eg.

Funding: The present work was supported by Princess Nourah bint Abdulrahman University Researchers Supporting Project number (PNURSP2025R91), Princess Nourah bint Abdulrahman University, Riyadh, Saudi Arabia.

Conflicts of Interest: The authors declare no competing interest in this study.

Keywords: Heart failure: dapagliflozin, polymorphism, cardiac fibrosis, Renal outcome, precision medicine.

Supplementary material

Table S1. Distribution of the studied genetic polymorphisms among study participants.

SNP	Minor allele Frequency (MAF*)	Genotype	Frequency	Percent	HWE‡
SLC5A2 rs3813008	MAF = 0.41	GG	63	31.5%	0.08
		GA	109	54.5%	
		AA	28	14%	
KCNJ11 rs5219	MAF = 0.24	CC	119	59.5%	0.18
		CT	66	33%	
		TT	15	7.5%	
UMOD rs12917707	MAF = 0.33	GG	95	47.5%	0.14
		GT	79	39.5%	
		TT	26	13%	
ACE rs4343	MAF = 0.46	AA	62	31%	0.37
		AG	93	46.5%	
		GG	45	22.5%	

*Minor allele frequencies obtained from the 1000 Genome, Phase 3 data (http://phase3browser.1000genomes.org/index.html).
A, G, C, T (Adenine, guanine, cytosine, thymine); SNP, Single Nucleotide Polymorphism; HWE, Hardy Weinberg equilibrium.

Table S2. Renal parameters and biochemical markers comparison among different genotypes for rs3813008, rs5219, rs129177 and rs4343.

**Genotype**	**eGFR (ml) after 6 months** Mean±SD	**Change in eGFR (ml)** Mean±SD	**KIM-1 (pg/ml) after 6 months** Mean±SD	**Change in KIM-1 (pg/ml)** Mean±SD	**NGAL (pg/ml) after 6 months** Mean±SD	**Change in NGAL (pg/ml)** Mean±SD
SLC5A2 SNP rs3813008						
GG	60.4 ± 23.9	-2.6 ± 3.6	190.2 ± 58.9	43.2 ± 33.6	266.6 ± 71.2	73.3 ± 18.5
GA	75.5 ± 24.3	2.9 ± 4.7	186.5 ± 87.2	21.4 ± 48.3	239.1 ± 26.5	10.9 ± 19.8
AA	75.1 ± 23.1	7.2 ± 6.5	171.8 ± 81.6	-31.7 ± 49.1	247.9 ± 35.9	-6.1 ± 15.4
Test Statistic	F=5.6	F=26.7	F=0.62	F=18.2	F=0.82	F=24.8
P-Value	0.004*	<0.0001*	0.54	<0.0001*	0.44	<0.0001*
KCNJ11 SNP rs5219						
CC	70.2 ± 31.5	1.3 ± 2.9	180.4 ± 40.7	17.9 ± 36.8	244.3 ± 48.6	31.4 ± 17.9
CT	72.2 ± 23.9	1.9 ± 2.6	197.8 ± 90.3	20.1 ± 38.3	263.9 ± 22.3	31.2 ± 26.7
TT	65.1 ± 23.2	4.3± 3.7	217.2 ± 83.4	12.9 ± 46.1	220.1 ± 29.7	-9.9 ± 25.2
Test Statistic	F=0.31	F=1.2	F=0.68	F=0.55	F=0.81	F=2.5
P-Value	0.74	0.32	0.51	0.58	0.45	0.09
UMOD SNP rs12917707						
GG	63.9 ± 32.1	0.87 ± 1.6	198.7 ± 95.4	33.6 ± 15.3	246.8 ± 48.2	36.7 ± 18.2
GT	76.2 ± 23.5	1.4 ± 2.8	182.9 ± 81.3	20.9 ± 10.4	264.9 ± 26.4	33.5 ± 27.6
TT	75.1 ± 23.9	6.3 ± 3.7	174.1 ± 80.4	-13.6 ± 18.6	208.9 ± 61.6	-19.4 ± 24.9
Test Statistic	F=4.1	F=6.3	F=0.36	F=4.7	F=1.7	F=7.5
P-Value	0.017*	<0.002*	0.70	0.01*	0.19	0.001*
ACE SNP rs4343						
AA	62.7 ± 21.7	3.9 ± 2.7	158.2 ± 89.5	30.1 ± 13.5	212.8 ± 33.9	9.4 ± 7.1
AG	76.1 ± 32.6	0.31 ± 1.9	194.1 ± 70.3	-26.2 ± 17.1	281.4 ± 39.1	-42.7 ± 6.8
GG	78.0 ± 24.3	-0.71 ± 1.2	218.9 ± 84.1	-37.3 ± 11.8	279.3 ± 18.6	-46.9 ± 6.3
Test Statistic	F=5.7	F=9.1	F=6.4	F=10.6	F=6.5	F=6.7
P-Value	0.004*	<0.0001*	0.002*	<0.0001*	0.002*	0.002*

Statistical analysis: Values are represented as means ± SD; (*) significantly different from baseline values is at P < 0.05. eGFR, estimated Glomerular filtration rate; KIM-1, Kidney Injury Molecule -1; NGAL, Neutrophil Gelatinase-Associated Lipocalin.

Table S3. Logistic Regression Analysis of SNPs and Clinical Predictors of Treatment Response.

**Predictors of Response**	**β coefficient**	***p*-Value**	**Odds Ratio**	**95%CI for Exp(B)**
rs3813008	1.922	3 E06 *	6.8	2.9 - 15.7
rs5219	0.097	0.782	1.1	0.54 - 2.2
rs1291770	0.779	0.011*	2.2	1.2 - 4.1
rs4343	-0.204	0.498	0.82	0.45 -1.47
Age	0.012	0.607	1.01	0.96 -1.06
Sex	0.646	0.304	1.9	0.56 - 6.5
Diabetes	0.448	0.363	1.56	0.59 - 4.1
Hypertension	0.246	0.628	1.28	0.47 -3.4
Serum Creatinine	1.964	0.254	7.13	0.24 - 2.8
Baseline eGFR	0.021	0.467	1.02	0.96 -1.07
BUN	0.004	0.899	1.04	0.94 -1.06

BUN: Blood Urea Nitrogen, eGFR: estimated glomerular filtration rate, CI: confidence interval. *Rs3813008, rs5219, rs1291770 and rs4343* genotypes were included as predictors with three levels (coded as 0, 1 and 2). Clinical variables with p – value < 0.20 were tested in the model.; *: significant difference ($p < 0.0125$).

Table S4. Associations of SNPs with Baseline Kidney Parameters and Changes Over Time Post-SGLT2i.

**SNP**	**Model 1 Baseline eGFR**		**Model 2 Change in eGFR**		**Model 3 Baseline KIM-1**		**Model 4 Change in KIM-1**		**Model 5 Baseline NGAL**		**Model 6 Change in NGAL**	
	β (S.E)	P-value	β (S.E)	P-value	β (S.E)	P-value	β (S.E)	P-value	β (S.E)	P-value	β (S.E)	P-value
rs3813008	9.1 (3.2)	0.003*	5.1 (0.7)	0.0001*	0.18 (0.87)	0.035*	-0.276 (0.05)	0.00003*	17.6 (6.9)	0.031*	-23.7 (6.9)	0.00007*
rs5219	1.92 (3.4)	0.582	0.86 (0.48)	0.29	0.15 (0.9)	0.19	0.1 (0.05)	0.92	9.6 (3.7)	0.45	-9.9 (7.9)	0.21
rs1291770	7.6 (2.9)	0.001*	1.98 (0.72)	0.006*	0.57 (0.22)	0.009*	-0.12 (0.05)	0.021*	7.43 (3.9)	0.51	-19.7 (6.9)	0.005

Rs3813008, rs5219, rs1291770 and rs4343 genotypes were included as predictors with three levels (coded as 0, 1 and 2). Clinical variables with p – value < 0.20 were tested in the model. Variables were retained in the model if they achieved statistical significance ($p \leq 0.05$). CI: confidence interval.

Table S5. Multivariate regression analysis for predictors of variability in clinical response to Dapagliflozin therapy among HFrEF patients.

**Predictors**	**Model 1 Change in eGFR**		**Model 2 Change in KIM-1**		**Model 3 Change in NGAL**	
	β (S.E)	P-value	β (S.E)	P-value	β (S.E)	P-value
rs3813008	4.6 (0.77)	<0.0001*	-0.26 (0.05)	<0.001	-0.42 (0.76)	<0.001
rs1291770	1.59 (0.78)	0.01*	2.92 (1.08)	<0.001	-0.16 (0.77)	0.032
Baseline KIM-1	------	------	0.19 (0.04)	<0.001	------	------
Intercept	-2.2	0.001	0.16	0.018	7.6	0.004
R^2	0.48		0.24		0.23	

Rs3813008, rs5219, rs1291770 and rs4343 genotypes were included as predictors with three levels (coded as 0, 1 and 2). Clinical variables with p – value < 0.20 were tested in the model. Variables were retained in the model if they achieved statistical significance (p ≤ 0.05). BUN: Blood Urea Nitrogen, eGFR: estimated glomerular filtration rate, CI: confidence interval.

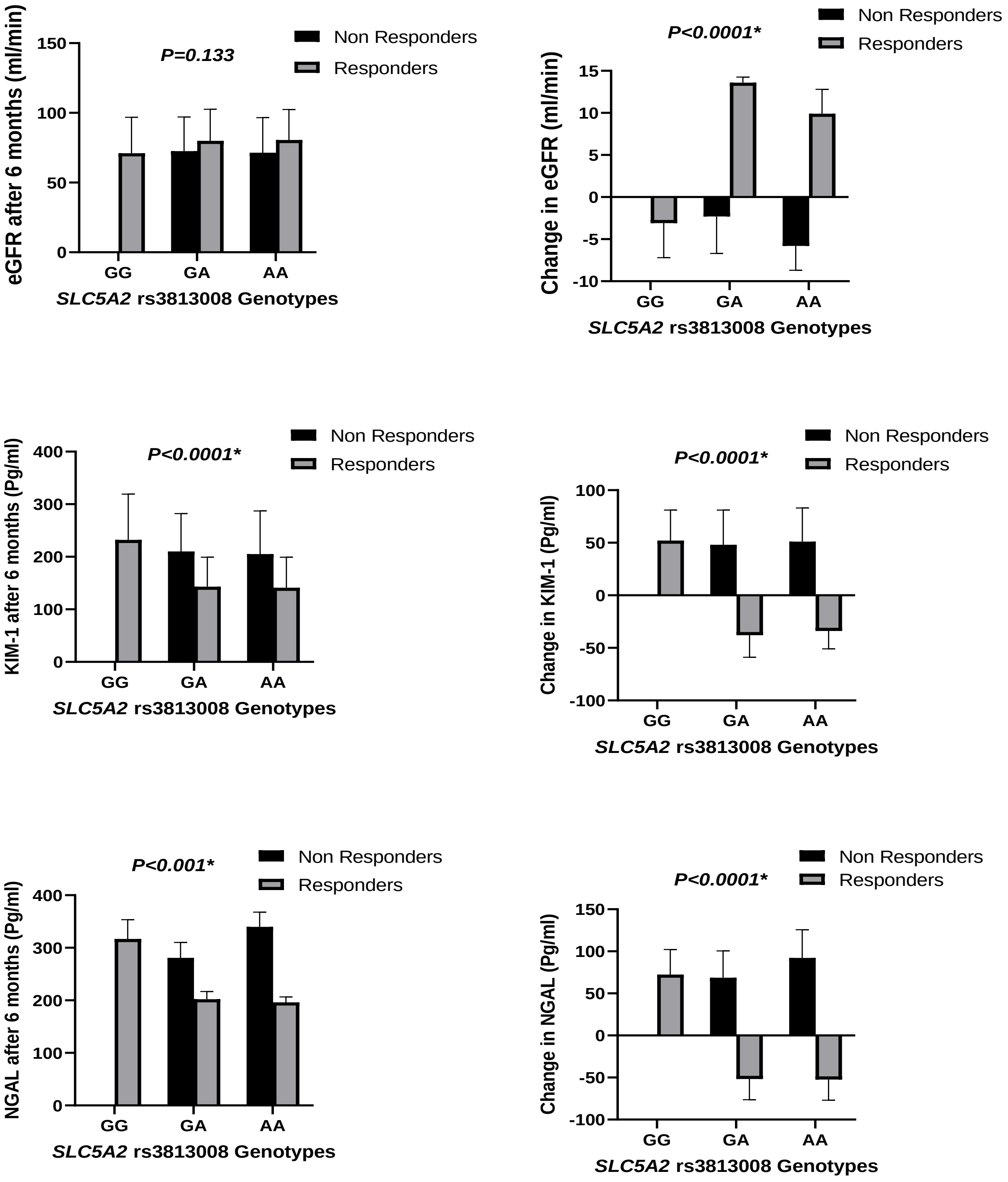

Figure S1. Association Between rs3813008 (SLC5A2) Genotypes and Renal Outcomes. This figure illustrates the impact of rs3813008 (SLC5A2) genetic polymorphisms on renal outcomes in patients treated with dapagliflozin. Individuals with the AA genotype exhibited the greatest improvement in eGFR and the most significant reductions in KIM-1 and NGAL levels, suggesting a protective effect of the A allele in renal function preservation. eGFR, estimated Glomerular filtration rate; KIM-1, Kidney Injury Molecule -1; NGAL, Neutrophil Gelatinase-Associated Lipocalin.S.

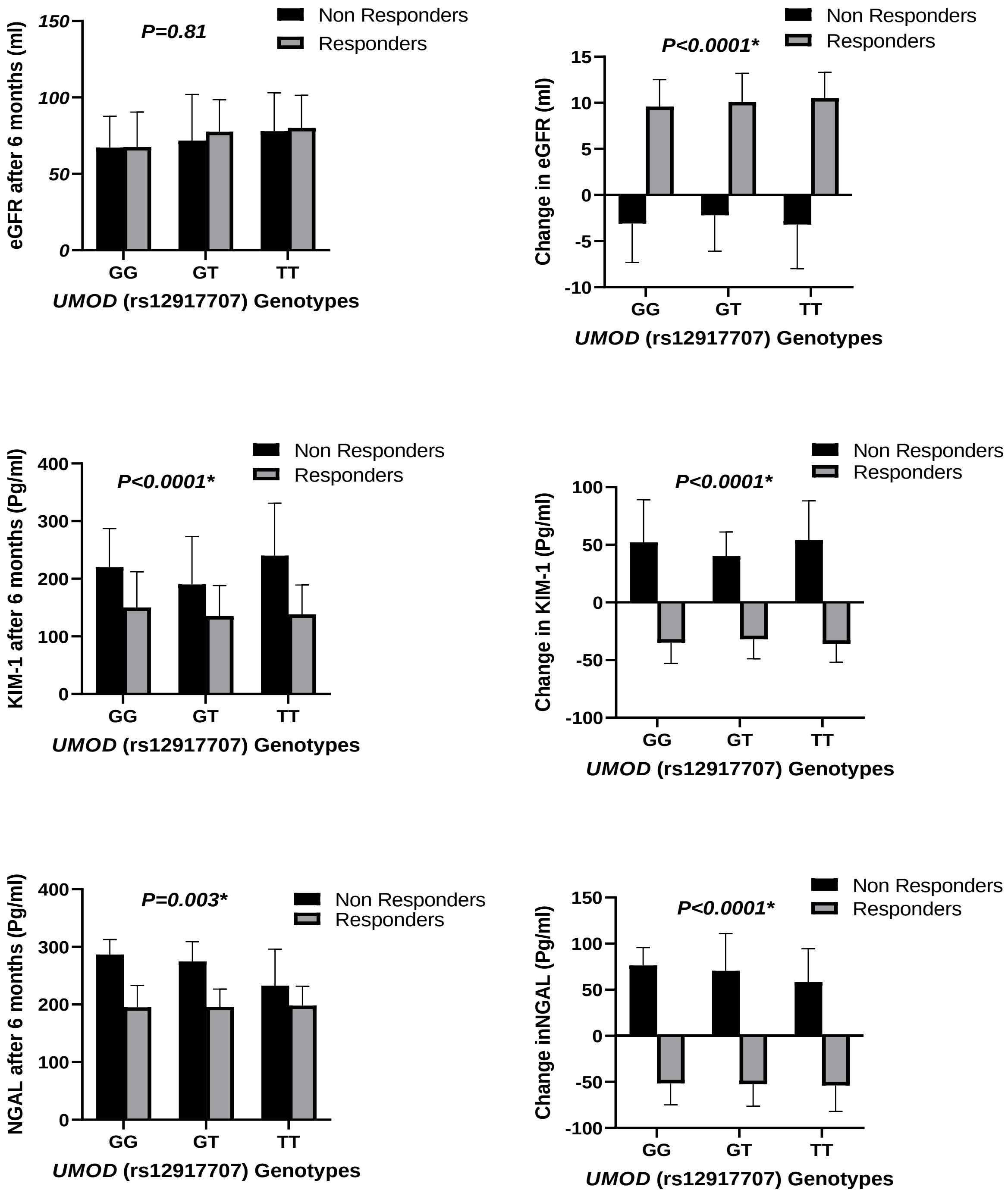

Figure S2. Association Between rs12917707 (*UMOD*) Genotypes and Renal Outcomes. This figure depicts the relationship between rs12917707 (UMOD) genotypes and renal response to dapagliflozin. Patients with the TT genotype demonstrated significant improvements in eGFR and reductions in KIM-1 levels, indicating a potential protective role of this variant in renal function. eGFR, estimated Glomerular filtration rate; KIM-1, Kidney Injury Molecule -1; NGAL, Neutrophil Gelatinase-Associated Lipocalin.

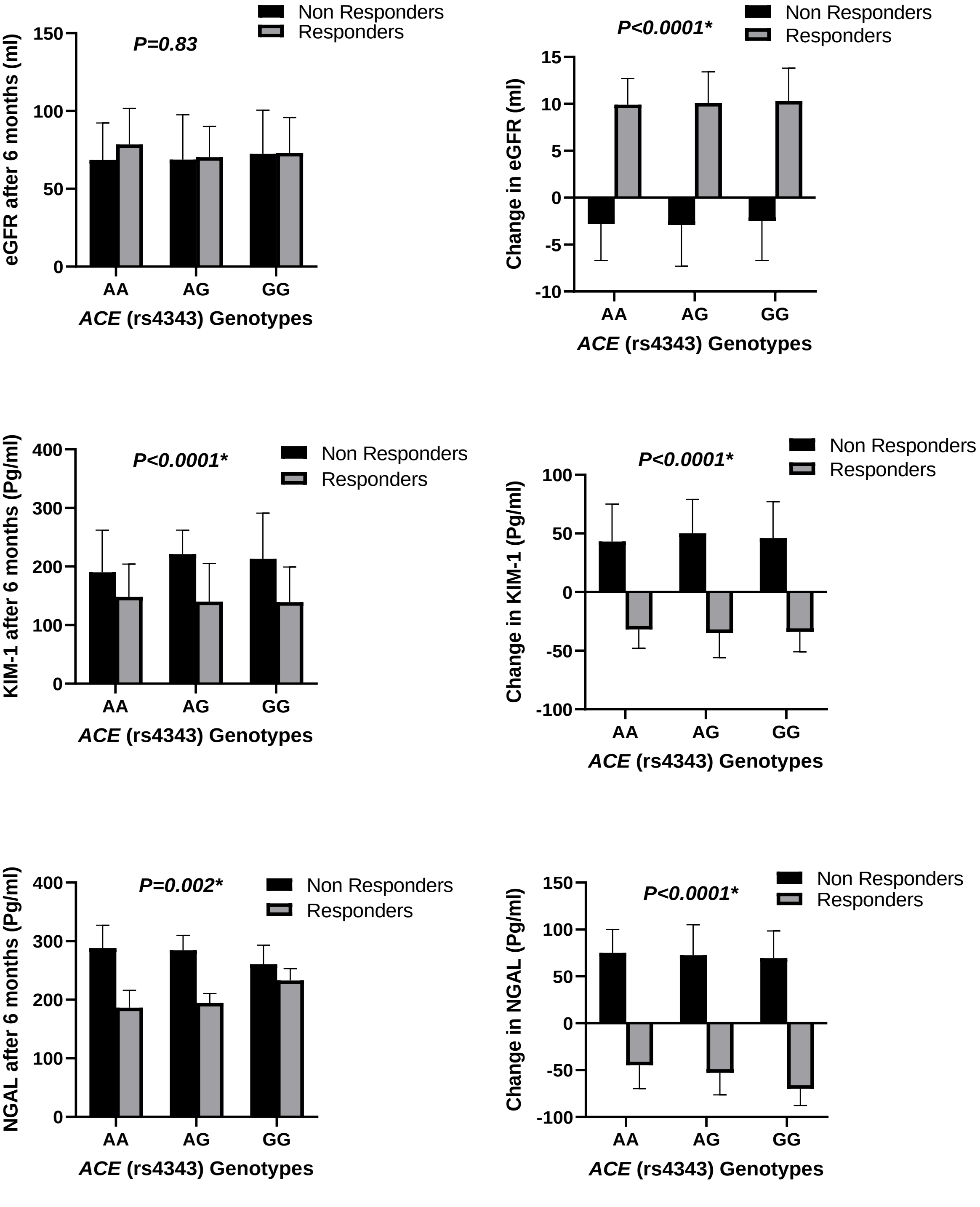

Figure S3. Association Between rs4343 (*ACE*) Genotypes and Renal Outcomes. This figure presents the effect of rs4343 (ACE) genetic variation on renal response to dapagliflozin. The GG genotype was associated with the most significant increase in eGFR and substantial decreases in both KIM-1 and NGAL levels, highlighting the influence of ACE polymorphisms on renal protective therapy. eGFR, estimated Glomerular filtration rate; KIM-1, Kidney Injury Molecule -1; NGAL, Neutrophil Gelatinase-Associated Lipocalin.

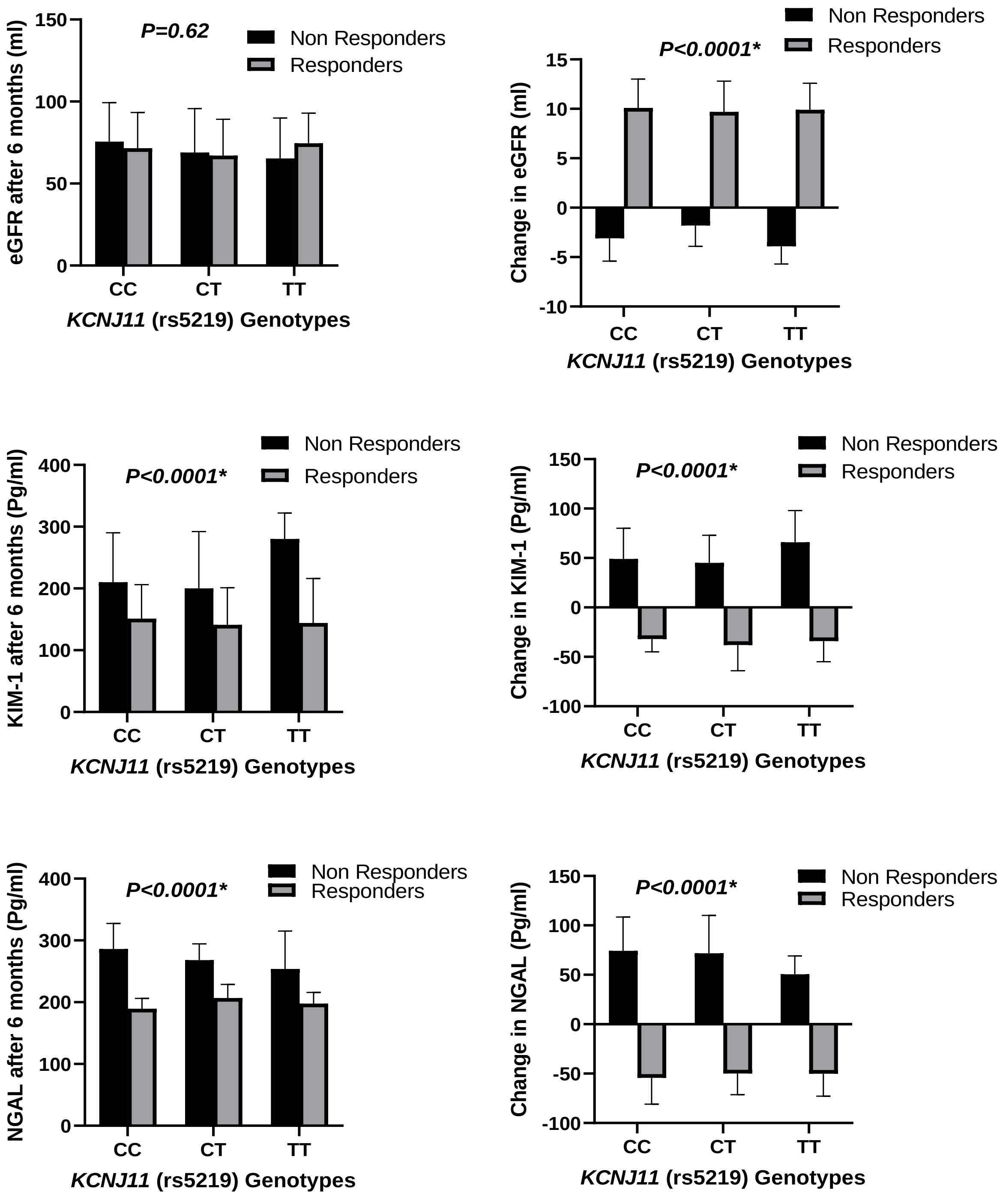

Figure S4. Lack of Association Between rs5219 (*KCNJ11*) Genotypes and Renal Outcomes. This figure shows the comparison of renal parameters across rs5219 (KCNJ11) genotypes. No significant differences were observed in eGFR, KIM-1, or NGAL levels among different genotype groups, indicating that rs5219 does not play a major role in modulating renal responses to dapagliflozin in this cohort. eGFR, estimated Glomerular filtration rate; KIM-1, Kidney Injury Molecule -1; NGAL, Neutrophil Gelatinase-Associated Lipocalin.
